# Long-term respiratory follow-up of H1N1 infection

**DOI:** 10.1186/1743-422X-8-319

**Published:** 2011-06-25

**Authors:** Paul Zarogoulidis, George Kouliatsis, Nikolaos Papanas, Dionysis Spyratos, Theodoros C Constantinidis, Ioannis Kouroumichakis, Paschalis Steiropoulos, Maria Mabroudi, Dimitris Matthaios, Theodora Kerenidi, Nikolaos Courcoutsakis, Konstantinos Zarogoulidis, Efstratios Maltezos

**Affiliations:** 1 Unit of Infectious Diseases, Democritus University Thrace, Dragana, 68100 Alexandroupolis, Greece; 2 Intesive Care Unit, Democritus University Thrace, Dragana, 68100 Alexandroupolis, Greece; 3 Laboratory of Hygiene and Environmental Protection and Regional Laboratory of Public Health, Medical School, Democritus University of Thrace, Eastern Macedonia-Thrace, 68100 Alexandroupolis, Greece; 4 Pulmonary Department, Aristotle University of Thessaloniki, "G.Papanikolaou" General Hospital, Exohi, 57010 Thessaloniki, Greece; 5 Pulmonary Department, University of Thessaly, Volos 38221, Greece; 6 Department of Radiology, Democritus University of Thrace, Dragana, 68100 Alexandroupolis, Greece

## Abstract

**Background:**

The first case of 2009 pandemic influenza A (H1N1) virus infection was documented in our Hospital on 10th August 2009.

**Metdods and findings:**

Real-time reverse-transcriptase-polymerase-chain-reaction (RT-PCR) testing was used to confirm the diagnosis. All patients were treated with oseltamivir from the first day of hospitalization. Upon admission 12/44 had local patchy shadowing in their chest x-ray and additionally antibiotic regimen was added to these patients as pneumonia was suspected based on clinical evidence. In total 44 patients were hospitalized 15/44 had asthma, 6/44 COPD, 5/44 leukemia. Lung function was evaluated with forced vital capacity, forced expiratory volume in 1 sec and diffused carbon monoxide upon discharge and every 3 months, until 6 months of observation was completed after discharge. The purpose of this retrospective cohort study was to evaluate whether influenza A (H1N1) had an impact on the respiratory capacity of the infected patients.

**Conclusions:**

An improvement of pulmonary function tests was observed between the first two measurements, implicating an inflammatory pathogenesis of influenza A (H1N1) to the respiratory tract. This inflammation was not associated with the severity or clinical outcome of the patients. All patients had a mild clinical course and their respiratory capacity was stable between the second and third measurement, suggesting that the duration of respiratory inflammation was two months. Early treatment with antiviral agents and vaccination represent the mainstay of management.

## Introduction

The first human infections with the new influenza A (H1N1) virus were confirmed in April 2009 in America, but the infection had been rapidly spreading around the world and in June 2009 World Health Organization (WHO) declared a pandemic [1-5]. WHO had advised countries in the northern hemisphere to prepare for a second wave of pandemic spread [6]. To date, almost all countries in the world have confirmed a second wave of H1N1 virus [7,8]. For this reason a working group of WHO member states agreed on a pandemic influenza preparedness framework [9]. The reason for creating this framework began with the need for real time virological data exchange among laboratories in different countries upon a pandemic outbreak [10,11]. The H1N1 virus has resulted from a triple recombination containing genes from human, avian, and swine influenza virus [12,13]. The incidence, clinical characteristics and factors affecting the patients outcome of the first wave have been well described in previous published studies [14-17]. However, while the numbers of positive H1N1 patients were lower, the hospitalisation rates were higher in comparison to the numbers and rates of 2009. In addition, the proportion of hospitalised cases admitted to intensive care units (ICU) was higher in 2010. The reason for these differences are not yet completely clear. There has been no obvious change in the severity of pandemic influenza A (H1N1) 2009 disease thresholds for hospital and ICU admission [8]. Three possible explanations were given. First, a new pandemic influenza A (H1N1) genetic variant predominated in the winter 2010 influenza season [7]. The Influenza A (H1N1) 2009 strain had undergone mutation in the hemagglutin. This mutation was associated with severe clinical outcome in comparison to the usual influenza A (H1N1) 2009 strain [7,18,19]. Secondly, resistance to oseltamivir developing from the first wave reached a peak in the 2010 pandemic [20,21]. Thirdly, it has been reported that this year's seasonal vaccine effectiveness was moderate, suggesting an insufficient protective effect against the second wave of influenza A (H1N1) [22-27].

In this retrospective study, we evaluated the respiratory capacity of 44 patients admitted to the Unit of Infectious Diseases (UID) of our hospital from 10^th ^August 2009 to 15^th ^November 2010. These were followed with Forced Vital Capacity (FVC), Forced Expiratory Volume in 1 sec (FEV1) and Carbon Monoxide Diffusing Capacity (DLCO) for a period of six months after their discharge. The rationale for the study was published data that H1N1 presents with hypoxemia and acute respiratory distress syndrome (ARDS) [14-17,28,29]. In addition, we examined the clinical characteristics of these patients.

## Patients and methods

### Methods of establishing H1N1 and precaution measures

Pharyngeal or nasopharyngeal swabs were taken upon admission in accordance with the protocol from the U.S. Centers for Disease Control and Prevention, as recommended by WHO and the average time between obtaining the samples and testing was 8-48 hours. The laboratory of the University Hospital of Alexandroupolis is a reference center for infectious diseases for more than 350.000 residents and one of the most important reference centers in Nothern Greece. Confirmed case was defined by a positive result of a real-time reverse-transcriptase-polymerase-chain-reaction (RT-PCR) [30].

The pandemic (H1N1) 2009 influenza virus is thought to spread from person to person in the same way as seasonal influenza, where transmission occurs predominantly through droplets produced from coughing or sneezing. Indirect transmission may also occur through self-inoculation after contact with surfaces or objects contaminated with the virus from infected persons. The incubation period for pandemic (H1N1) 2009 influenza virus is understood to be approximately four days (range: 1-7 days). The period of communicability is estimated to be seven days in uncomplicated cases; however, it may be longer in children (up to 10 days) and other individuals in whom symptoms and virus shedding may persist (i.e. immuno-compromised and severely ill). Consistent with seasonal influenza, transmission of the pandemic (H1N1) 2009 influenza virus is most likely during the initial days of infection when cases are typically symptomatic and have higher viral loads [31].

The following isolation precautions were recommended for healthcare personnel who are in close contact with patients with suspected or confirmed 2009 H1N1 influenza. Close contact was defined as working within 6 feet of the patient or entering into a small enclosed airspace shared with the patient (e.g. average patient room). The standard precaution for all patient care was use of non-sterile gloves for any contact with potentially infectious material, followed by hand hygiene immediately after glove removal; use of gowns along with eye protection for any activity that might generate splashes of respiratory secretions or other infectious material.

Respiratory protection recommendation, according to CDC continues to be the use of respiratory protection that is at least as protective as a fit-tested disposable N95 respirator for healthcare personnel who are in close contact with patients with suspected or confirmed 2009 H1N1 influenza. This recommendation applies uniquely to the special circumstances of the H1N1 pandemic during the fall and winter of 2009-2010 and CDC will continue to revisit its guidance as new information becomes available, within this season if necessary.

The current recommendation is based on the unique conditions associated with the current pandemic, including low levels of population immunity to 2009 H1N1 influenza, availability of vaccination programs well after the start of the pandemic, susceptibility to infection of those in the age range of healthcare personnel, increased risk for complications of influenza in some healthcare personnel (e.g. pregnant women), and the potential for healthcare personnel to be exposed to H1N1 influenza patients because of their occupation [32].

Respiratory evaluation with FVC, FEV1 and DLCO was determined according to a joint statement based on the previous statements from the American Thoracic Society and European Respiratory Society [33]. Patients were evaluated the day of their discharge and every three months until six months all follow up were complete for each one. The concept was to evaluate whether these patients had clinical presentation of adverse effects of the virus on their respiratory capacity and to establish the duration of these effects.

### Statistical analysis

Analysis was carried out with the use of SPSS statistical software package (SPSS version 17.01; SPSS, Chicago, IL, USA). Continuous variables were expressed as mean ± SD or median (with interquartile ranges). For categorical variables, the percentages of patients in each category were calculated. Unpaired t-test was used in normally distributed parameters to compare the mean values between the two groups. A p value of less than 0.05 was considered to indicate statistical significance.

## Results

Mean patient age was 36 years (14-65). The majority of the confirmed patients hospitalized were Caucasian male (28/44). All patients were treated immediately with oseltamivir (75-150 mg every 12 hours). Treatment with oseltamivir was continued for 4 to 10 days depending on the individual patient's clinical condition. In 12/44 patients, empiric antibiotic treatment (azithromycin+amoxicillin+clavulanic-acid or ceftriaxone+quinolone) was given upon admission due to suspected secondary bacterial pneumonia elevated C-reactive protein, WBC count and local patchy shadowing on x-ray as reported in previous studies [34-36]. C-reactive protein and WBC count were elevated in these patients with local patchy shadowing on radiography (12/44 pts 27.2%).

Patients with radiological evidence of pneumonia were tested for serum procalcitonin (PCT) (36 patients in total) and Legionella/Streptococcus pneumoniae urinary antigens. Pneumonia score index (PSI) was applied and empirical antibiotic treatment was initiated according the treating physician's clinical judgment. Patient's procalcitonin test was within normal range (0.05-0.1) in all patients. Additionally, urine antigen results for Legionella and Streptococcus pneumoniae and blood culture that were drawn were also negative. The pneumonia score index was evaluated in these patients and the range was between class II to IV for 12/44 patients [37].

Coexisting conditions were as follows: asthma 15/44 (34%), chronic obstruction disease 6/44 (13.6%), diabetes mellitus 8/44 (18.1%), coronary heart disease 10/44 (22.7%), lymphoma 5/44 (11.3%). Median laboratory values were: C- reactive protein 5.18 mg/dl, white blood count (WBC) 7.114, urea 26.18 mg/dl, creatinine 0.9 mg/dl, aspartate aminotransferase (AST) 27.09 IU/l, alanine aminotransferase (ALT) 24.48 IU/l. Abnormal liver function was observed in 7/44 (15.9%). Mean temperature and oxygen saturation upon admission were 39.06°C and 96.07%, respectively. Median PO_2 _was 81.23 mmHg. Severe hypoxemia upon admission, defined as decreased partial pressure of oxygen in blood ≤ 60 mmHg with FiO_2 _21% [29], was observed in 7/44 patients. There was no thrombocytopenia observed. Median duration of hospitalisation and fever were 5.39 days and 2.45 days, respectively. Median body-mass index (BMI) was 30.23 kg/m^2^. Obesity (BMI ≥ 30 kg/m^2 ^[38]) was noted in 11/44 and morbid obesity (BMI ≥ 40 kg/m^2^) was noted in 12/44 patients. Patient characteristics are presented in Table 1.

**Table 1:** Patients characteristics

	Upon admission			Upon Discharge		
**Characteristic H1N1(+)**	**Mean**	**Range**	**(±SD)**	**Mean**	**Range**	**(±SD)**

**Age (years)**	36	14-65	(14.7)			

						

**Male/Female (n, %)**	28/16 (63.6%/36.3%)					

						

**Smokers**						

(male/female) (n, %)	8/28 (28.6%) and 3/16 (18.8%)					

						

**Non-Smokers**						

(male/female)	20/28 (71.4%) and 13/16 (81.3%)					

						

**BMI**	30.23	20-45	(8.757)			

No,pts with obesity (male/female)	7/28 (25%) and 4/16 (25%)					

No.pts without obesity (male/female)	21/28 (75%) and 12/16 (75%)					

**Co-exististing conditions (n, %)**						

Asthma	15/44 (34%)					

COPD	6/44 (13.6%)					

IPF	1/44 (2.2%)					

Lymphoma	5/44 (11.3%)					

Diabetes	8/44 (18.1%)					

Coronary Heart Disease	10/44 (22.7%)					

						

**Outcomes-days**						

Duration of fever in hospital under treatment	2.45	1-4	(0.875)			

Days of Hospitalization	5.39	3-12	(1.781)			

Days under oseltamivir regimen	5.25	4-10	(1.014)			

						

**Adverse events (n, %)**						

Abnormal liver function	7/44 (15.9%)					

Nausea, Diarrhea	4.5%					

Vomiting	1.4%					

Hypoxemia	1/44 (21.2%)					

CRP	5.18	0.26-17.38	(5.123)	1.33	0.16-4.53	(1.01)

WBC	7.114	3.340-10.950	(2172.160)	6.806	3340-10950	(2021.6)

Fever	39.06	37-40	(0.834)	37.8	36.3-39.2	(0.77)

CR	0.9	0.5-1.30	(0.17)	0.8	5-1	(0.12)

UR	26.18	11-41	(7.469)	25.11	12-40	(7.127)

SGPT	24.48	10-138	(26.37)	27.80	9-136	(26.719)

SGOT	27.09	14-125	(22.941)	24.14	9-57	(12.22)

Spo_2 _(FiO_2 _21%)	96.07	89-99	(2.3)	96.98	94-99	(1.248)

PO_2 _(FiO_2_21%)	81.23	53-113	(14.586)	87.66	67-113	(12.473)

						

**Abnormalities on chest radiograph (n, %)**						

Local patchy shadowing	8/44 (18.1%)			6/44 (13.6%)		

Ground-glass opacities	3/44 (6.8%)			2/44 (4.5%)		

Interstitial abnormality	1/44 (2.2%)			1/44 (2.2%)		

No further pharyngeal or nasopharyngeal swabs were taken during hospitalization or after treatment. The criteria for discharge were absence of hypoxemia and temperature ≤ 37°C for 1 day without antipyretic treatment [17].

**Figure 1:**
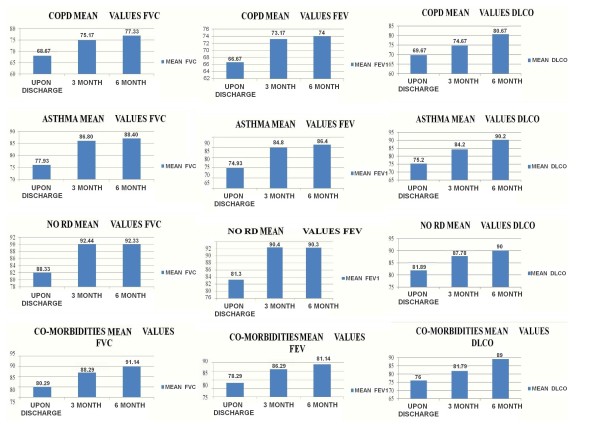
**Forced Vital Capacity (FVC), Forced Expiratory Volume in 1 sec (FEV1), Carbon Monoxide Diffusing Capacity (DLCO) mean values for patients with Chronic Obstructive Pulmonary Disease (COPD), patients with Asthma, patients without any respiratory disease (NO RD), patients with co-morbidities**.

Patient's respiratory capacity was evaluated with FVC, FEV1 and DLCO upon discharge and every three months until six months of observation was completed for each patient. The evaluation is demonstrated according to four categories which the authors selected based on the same underlying disease of the patients: a) chronic obstructive pulmonary disease, b) asthma, c) co-morbidities (diabetes mellitus, coronary heart disease, and cancer), d) previously healthy patients. Whenever a patient had both an underlying respiratory disease and co-morbidity he was selected to be included in a group according to his major factor affecting his clinical outcome, preferably his underlying respiratory background. There was no association between radiographic data and pulmonary function tests. The mean values for FVC, FEV1 and DLCO are presented in Figures 1. A significant difference was observed between mean values of FEV1, FVC and DLCO between the evaluations upon discharge and the sixth month follow up (Table 2).

**Table 2:** Statistical findings as mean values between follow ups.

**DLCO**	1DLCO	76.07	-6.750	.000
	2DLCO	82.82		
	1DLCO	76.07	-12.409	.000
	3DLCO	88.48		
	2DLCO	82.82	-5.659	.000
	3DLCO	88.48		
**FEV1**	1FEV1	76.18	-8.659	.000
	2FEV1	84.84		
	1FEV1	76.18	-10.205	.000
	3FEV1	86.39		
	2FEV1	84.84	-1.545	.001
	3FEV1	86.39		
**FVC**	1FVC	78.52	-8.318	.000
	2FVC	86.84		
	1FVC	78.52	-9.909	.000
	3FVC	88.43		
	2FVC	86.84	-1.591	.000
	3FVC	88.43		

## Discussion

We described a retrospective cohort study of 44 patients who were hospitalized with the pandemic 2009/10 Influenza A (H1N1) in the University Hospital of Alexandroupolis, Thrace from 10^th ^August 2009 to 15^th ^November 2010. So far, there has been no evaluation of H1N1 respiratory function. The correlation between the mechanisms and the impact that viruses have on the respiratory system has been established in previous published studies [14-17,28,29,39-44]. As might be anticipated, we found a major decreased respiratory capacity (FEV1 -10.205, FVC -9.909, DLCO -12.409) between the measurements taken upon discharge and in the six-month follow-up. This occurred due to the inflammatory effects of the virus through the respiratory tract. The differentiation of respiratory capacity also varied between the measurements taken upon discharge and the three-month follow-up, but it was observed that the variation was smaller in comparison to the sixth-month visit, which is possibly due to the time needed for the resolution of the inflammatory effects.

Patients with previously diagnosed respiratory disease such as COPD and asthma were well controlled on their treatment. H1N1 induced inflammation and was the factor that dysregulated their condition. WBC count and CRP were elevated in patients with suspected bacterial infection. Hypoxemia was more severe in patients with opacities on chest x-rays. The mean values in 7/44 patients with abnormal liver functions did not exceed 138 IU/L for ALT and 125 IU/L for AST. These patients did not present jaundice or other clinical signs of liver dysfunction. Patients with BMI ≥ 30 kg/m^2 ^were 20/44, implicating a mild clinical course overall, since BMI ≥ 30 is considered an adverse prognostic factor. The laboratory findings were not correlated with the respiratory functions, based on the fact that patients upon exacerbation or with opacities on chest x-ray would present a severe deterioration of their respiratory capacity. None of the patients had been previously vaccinated, but all patients received oseltamivir immediately on hospitalization and antibiotics upon suspicion of bacterial respiratory infection preventing an escalation of the inflammation and infection.

The mechanisms of H1N1-induced respiratory effects have received considerable attention [39-44]. Recent studies have shown that the high mortality rate of avian influenza virus infections is a consequence of an overactive inflammatory response and the severity of infection is closely related with virus-induced cytokine dysregulation. The most important feature of influenza A immune-pathogenesis is the appearance of "cytokine storm", which is characterized by the extreme production and secretion of numerous pro-inflammatory cytokines. This is responsible for the development of lethal clinical symptoms, such as massive pulmonary edema, acute bronchopneumonia, alveolar hemorrhage, reactive hemophagocytosis, and acute respiratory distress syndrome, associated with necrosis and tissue destruction. Numerous in vitro, in vivo and clinical studies have pointed out that A/H5N1 viruses are very strong inducers of various cytokines and chemokines (Tumor Necrosis Factor [TNF]-alpha, Interferon [IFN]-gamma, IFN-alpha/beta, Interleukin [IL]-6, IL-1, MIP [Macrophage Inflammatory Protein] -1, MIG [Monokine Induced by IFN-gamma], IP [Interferon-gamma-Inducible Protein]-10, MCP [Monocyte Chemoattractant Protein]-1, RANTES [Regulated on Activation Normal T-cell Expressed and Secreted], IL-8), in both humans and animals. The major cells implicated in the cytokine storm are macrophages and CD8+ T-lymphocytes, while the primary contributor cytokines are TNF-alpha, IL-6 and IFN-gamma. It has been detected that mutations of some viral genes (NS1, PB2, HA and NA) are responsible for the "cytokine storm", by increasing the viral replication rate, expending the tissue tropism, as well as facilitating the systemic invasion and the development of resistance against the host antiviral response. Glu92 and Ala149 mutations and carboxyl-terminal ESEV/EPEV motif of NS1 protein have been implicated as determinants of virulence for A/H5N1 strains. In addition, Lys627 mutation in PB2 protein, polybasic aminoacid mutations in the cleavage region of hemagglutinin (HA) polyprotein, and glycosylation and sialyzation mutations in HA and neuraminidase (NA) proteins were found to enhance the immune-mediated pathology of highly virulent A strains [40-45]. Furthermore, in a preliminary analysis of macrophage gene expression data based on published studies, 64 genes in H1N1-infected cells showed at least 1.5-fold difference in expression level compared to mock infected cells in at least one time point [43]. Impressively, as many as 60 genes were thereby upregulated [43].

Given these important inflammatory processes, it is important to attempt patient prophylaxis. Several antiviral agents were tested both *in vitro *and/or *in vivo *and presented results implicating that the early use of such agents suppress the inflammation apart from being a therapy [43-49] Antibiotics especially macrolides, which are well known for their anti-inflammatory and immunomodulatory properties were also tested. Clarithromycin inhibits the middle to late stage of the influenza virus replication cycle, resulting in inhibition of progeny virus production from the infected cells. Macrolides could mediate this effect by inhibiting intracellular hemagglutinin HA0 proteolysis. The inhibitory effect on influenza virus replication of macrolides has been known since the 80s [50-54]. N-acetylcysteine (NAC) is a thiol-containing compound which non-enzymatically interacts and detoxifies reactive electrophiles and free radicals. NAC has already been shown to effectively protect human bronchial fibroblasts against the toxic effects of tobacco smoke condensates and the isolated perfused lung against the glutathione (GSH)-depleting effect of tobacco smoke. NAC has also been demonstrated to reduce the reactive oxygen intermediate hydrogen peroxide (H_2_O_2_) and to confer protection from the toxic effects of H_2_O_2_. In vivo studies, however, have demonstrated that orally administered NAC has very low bioavailability due to its rapid metabolism mainly to GSH but also other metabolites. Thus, even though NAC is very effective in protecting cells of different origins from the toxicity of reactive components in tobacco smoke and reactive oxygen species, a direct scavenging effect by NAC in vivo, does not seem likely. This holds especially true for oral administration. A more relevant mechanism in vivo for any protective effect of NAC may be attributable to its activity as a precursor of GSH, thereby facilitating its biosynthesis. GSH will then serve as the protective agent and detoxify reactive species both enzymatically and non-enzymatically [55,56]. Finally, the efficacy of vaccination for H1N1 has been documented by assessing antibody formation in the vaccinated elderly subjects from the 2009 pandemic in contrast to the younger ones who were not vaccinated. Nonetheless, antibodies from older subjects previous encounter with pandemic influenza A strains may be a confounding factor [57].

This study has a number of limitations. First, our patient series was small, but it should be borne in mind that since these H1N1 positive patients represent a population of 350.000 residents. Furthermore, diagnostic tests with procalcitonine, urine antigen and blood cultures were given only upon suspicion of an infection. The respiratory capacity tests represented a small number of patients in each group, and so no correlations could be preformed between the four subgroups. In addition, the baseline condition of each patient was not evaluated; this was either because they did not have a respiratory underlying condition or because they were patients that admitted in our hospital for the first time.

## Conclusions

Respiratory capacity differed between categories of patients at least for the first three months of observation, probably due to the inflammatory factors that are released with the influenza A (H1N1) infection and underlying disease. Importantly, the respiratory inflammation lasted almost two months, as evidenced by the fact that respiratory capacity remained stable between the second and third measurement. Several treatment modalities can be administered to confer protection and suppress the inflammation. Vaccination and early treatment with antiviral agents represent the mainstay of management.

## Conflicts of interests

The authors declare that they have no competing interests.

## Authors' contributions

PZ, DS, NP, MM and TK wrote the manuscript and treated the patients, TCC performed statistical analysis, GK, IK, DM, and PS performed lung function tests, NC evaluated the chest x-rays and CT scan when necessary, KZ and EM provided useful insight. All authors read and approved the manuscript

## References

[B1] Centers for Disease Control and Prevention (CDC) Update: swine influenza A (H1N1) infections--California and Texas, April 2009 MMWR Morb Mortal Wkly Rep 2009 58 16 435 437 19407739

[B2] Centers for Disease Control and Prevention (CDC) Update: novel influenza A (H1N1) virus infection--Mexico, March-May, 2009 MMWR Morb Mortal Wkly Rep 2009 58 21 585 589 19498336 PMC5856103

[B3] Centers for Disease Control and Prevention (CDC) Update: novel influenza A (H1N1) virus infection--worldwide MMWRMorb Mortal Wkly Rep 2009 58 17 453 458 19444146

[B4] World Health Organization. Pandemic (H1N1) 2009--update 69 http://www.who.int/csr/don/2009_10_09/en/[accessed October 19, 2009]

[B5] Devaux I Kreidl P Penttinen P Salminen M Zucs P Ammon A ECDC influenza surveillance group; national coordinators for influenza surveillance Initial surveillance of 2009 influenza A(H1N1) pandemic in the european union and European economic area, April-September 2009 Euro Surveill 2010 15 49 pii: 19740 21163182 10.2807/ese.15.49.19740-en

[B6] Global Alert and Response (GAR) Preparing for the second wave: lessons from current outbreaksPandemic (H1N1) 2009 briefing note 9 http://www.who.int/csr/disease/swineflu/notes/h1n1_second_wave_20090828/en/index.html

[B7] Barr IG Cui L Komadina N Lee RT Lin RT Deng Y Caldwell N Shaw R Maurer-Stroh S A new pandemic influenza A(H1N1) genetic variant predominated in the winter 2010 influenza season in Australia, New Zealand and Singapore Euro Surveill 2010 15 42 pii: 19692 21034722 10.2807/ese.15.42.19692-en

[B8] Bandaranayake D Jacobs M Baker M Hunt D Wood T Bissielo A Macfarlane M Lopez L Mackereth G Huang Q The second wave of 2009 influenza A(H1N1) in the New Zealand, January-October 2010 Euro Surveill 2011 16 6 pii: 19788 21329643

[B9] Eurosurveillance editorial team Agreement on a pandemic influenza preparedness framework for the sharing of viruses and benefit sharing Euro Surveill 2011 16 16 pii: 19847 21527130

[B10] Casalegno JS Frobert E Escuret V Bouscambert-Duchamp M Billaud G Mekki Y Schuffenecker I Lina B Morfin F Valette M Beyond the influenza-like illness surveillance: The need for real-time virological data Euro Surveill 2011 16 1 pii: 19756 21223833

[B11] Harder KM Andersen PH Bæhr I Nielsen LP Ethelberg S Glismann S Molbak K Electronic real-time surveillance for influenza-like illness: experience from the 2009 influenza A(H1N1) pandemic in Denmark Euro Surveill 2011 16 3 pii: 19767 21262186

[B12] Novel Swine-Origin Influenza A (H1N1) Virus Investigation Team Dawood FS Jain S Finelli L Shaw MW Lindstrom S Garten RJ Gubareva LV Xu X Bridges CB Uyeki TM Emergence of a novel swine-origin influenza A (H1N1)virus in humans N Engl J Med 2009 360 25 2605 15 19423869 10.1056/NEJMoa0903810

[B13] Taubenberger JK Reid AH Lourens RM Wang R Jin G Fanning TG Characterization of the 1918 influenza virus polymerase genes Nature 2005 437 7060 889 93 10.1038/nature04230 16208372

[B14] Santa-Olalla Peralta P Cortes-García M Vicente-Herrero M Castrillo-Villamandos C Arias-Bohigas P Pachon-del Amo I Sierra-Moros MJ Surveillance Group for New Influenza A(H1N1) Virus Investigation and Control Team in Spain Risk factors for disease severity among hospitalized patients with 2009 pandemic influenza A(H1N1) in spain, April-December 2009 Euro Surveill 2010 15 38 pii: 19667 20929651 10.2807/ese.15.38.19667-en

[B15] Gubbels S Perner A Valentiner-Branth P Molbak K National surveillance of pandemic influenza A(H1N1) infection-related admissions to intensive care units during the 2009-10 winter peak in Denmark: two complementary approaches Euro Surveill 2010 15 49 pii: 19743 21163180 10.2807/ese.15.49.19743-en

[B16] Zarogoulidis P Constantinidis T Steiropoulos P Papanas N Zarogoulidis K Maltezos E Are there any differences in clinical and laboratory findings on admission between H1N1 positive and negative patients with flu-like symptoms? BMC Res Notes 2011 in press 10.1186/1756-0500-4-4 PMC3035198 21214902

[B17] Cao B Li XW Mao Y Wang J Lu HZ Chen YS Liang ZA Liang L Zhang SJ Zhang B Gu L Lu LH Wang DY Wang C National Influenza A Pandemic (H1N1) 2009 Clinical Investigation Group of China: Clinical features of the initial cases of 2009 pandemic influenza A (H1N1) virus infection in China N Engl J Med 2009 361 26 2507 17 10.1056/NEJMoa0906612 20007555

[B18] Kilander A Rykkvin R Dudman SG Hungnes O Observed association between the HA1 mutation D222G in the 2009 pandemic influenza A(H1N1) virus and severe clinical outcome, Norway 2009-2010 Euro Surveill 2010 15 9 pii: 19498 20214869 10.2807/ese.15.09.19498-en

[B19] Maurer-Stroh S Lee RT Eisenhaber F Cui L Phuah SP Lin RT A new common mutation in the hemagglutin of the 2009 (H1N1) influenza A virus PLoS Curr 2010 2 RRN1162 20535229 10.1371/currents.RRN1162 PMC2880458

[B20] Lackenby A Moran Gilad J Pebody R Miah S Calatayud L Bolotin S Vipond I Muir P Guiver M McMenamin J Reynolds A Moore C Gunson R Thompson C Galiano M Bermingham A Ellis J Zambon M Continued emergence and changing epidemiology of oseltamivir-resistant influenza A(H1N1)2009 virus, United Kindom, Winter 2010/11 Euro Surveill 2011 16 5 pii: 19784 21315056

[B21] Hurt AC Deng YM Ernest J Caldwell N Leang L Iannello P Komadina N Shaw R Smith D Dwyer DE Tramontana AR Lin RT Freeman K Kelso A Barr IG Oseltamivir-resistant influenza viruses circulating during the first year of the influenza A(H1N1)2009 pandemic in the Asia-Pacific region, March 2009 to March 2010 Euro Surveill 2011 16 3 pii: 19770 21262183

[B22] Granados A Goodman C Eklund L Pandemic influenza: using evidence on vaccines and antivirals for clinical decisions and policy making Eur Respir J 2006 27 661 663 10.1183/09031936.06.00017406 16585070

[B23] Savulescu C Jiménez-Jorge S de Mateo S Ledesma J Pozo F Casas I Larrauri A cycEVA Study Team: Effectiveness of the 2010/11 seasonal trivalent influenza vaccine in Spain: preliminary results of a case-control study Euro Surveill 2011 16 11 pii: 19820 21435330 10.2807/ese.16.11.19820-en

[B24] Puig-Barberà J 2010-2011 influenza seasonal vaccine, preliminary mid-season effectiveness estimates: reason for concern, confounding or are we following the right track? Euro Surveill 2011 16 11 pii: 19821 21435331 10.2807/ese.16.11.19821-en

[B25] Kissling E Valenciano M I-MOVE case-control studies team Early estimates of seasonal influenza vaccine effectiveness in Europe, 2010/11: I-Move, a multicentre case-control study Euro Surveill 2011 16 11 pii: 19818 21435329 10.2807/ese.16.11.19818-en

[B26] Steens A van der Hoek W Dijkstra F van der Sande M Influenza vaccine effectiveness, 2010/11 Euro Surveill 2011 16 15 pii: 19843 21507318

[B27] Pebody R Hardelid P Fleming D McMenamin J Andrews N Robertson C Thomas D Sebastianpillai P Ellis J Carman W Wreghitt T Zambon M Watson J Effectiveness of seasonal 2010/11 and pandemic influenza A(H1N1)2009 vaccines in preventing influenza infection in the United Kindom: Mid-Season analysis 2010/11 Euro Surveill 2011 16 6 pii: 19791 21329644

[B28] Mauad T Hajjar LA Callegari GD da Silva LF Schout D Galas FR Alves VA Malheiros DM Auler JO Jr Ferreira AF Borsato MR Bezerra SM Gutierrez PS Caldini ET Pasqualucci CA Dolhnikoff M Saldiva PH Lung pathology in fatal novel human influenza A (H1N1) infection Am J Respir Crit Care Med 2010 181 1 72 9 Epub 2009 Oct 29 10.1164/rccm.200909-1420OC 19875682

[B29] Lee Warren L Slutsky Arthur S Mason Murray & Nadel's Textbook of Respiratory Medicine 4 Copyright ^© ^2005 Saunders, An Imprint of Elsevier21710137

[B30] CDC protocol of real-time RTPCR for swine influenza A (H1N1). Geneva: World Health Organization, April 28, 2009 http://www.who.int/csr/resources/publications/swineflu/CDCrealtimeRTPCRprotocol_20090428.pdf (AccessedNovember 30, 2009

[B31] MacIntyre CR Cauchemez S Dwyer DE Seale H Cheung P Browne G Fasher M Wood J Gao Z Booy R Ferguson N Face mask use and control of respiratory virus transmission in households Emerg Infect Dis 2009 15 2 233 41 10.3201/eid1502.081167 19193267 PMC2662657

[B32] Interim Guidance on Infection Control Measures for 2009 H1N1 Influenza in Healthcare Settings, Including Protection of Healthcare Personnel http://www.cdc.gov/flu/professionals/infectioncontrol/healthcaresettings.htm accessed: December 12, 2010 20066893

[B33] Miller MR Hankinson J Brusasco V Burgos F Casaburi R Coates A Crapo R Enright P van der Grinten CP Gustafsson P Jensen R Johnson DC MacIntyre N McKay R Navajas D Pedersen OF Pellegrino R Viegi G Wanger J ATS/ERS Task Force: Standardisation of spirometry Eur Respir J 2005 26 2 319 338 10.1183/09031936.05.00034805 16055882

[B34] Flood RG Badik J Aronoff SC The Utility of Serum C-Reactive Protein in Differentiating Bacterial from Nonbacterial Pneumonia in Children: A Meta-Analysis of 1230 Children Infect Dis J 2008 27 95 99 10.1097/INF.0b013e318157aced 18174874

[B35] George EL Panos A Does a high WBC count signal infection? Nursiing 2005 35 1 20 2 10.1097/00152193-200501000-00014 15622174

[B36] Ortega-Deballon P Radais F Facy O d'Athis P Masson D Charles PE Cheynel N Favre JP Rat P C-Reactive protein is an early predictor of septic complications after elective colorectal surgery World Journal of Surgery 2010 34 4 808 14 10.1007/s00268-009-0367-x 20049435 PMC2877195

[B37] Fine MJ Auble TE Yealy DM Hanusa BH Weissfeld LA Singer DE Coley CM Marrie TJ "A prediction rule to identify low-risk patients with community-acquired pneumonia" N Engl J Med 1997 336 4 243 250 10.1056/NEJM199701233360402 8995086

[B38] WHO Global Database on Body mass Index http://apps.who.int/bmi/index.jsp?introPage=intro_3.html

[B39] Belser JA Zeng H Katz JM Tumpey TM Infection with highly pathogenic H7 influenza viruses results in an attenuated proinflammatory cytokine and chemokine response early after infection J Infect Dis 2011 203 1 40 8 10.1093/infdis/jiq018 21148495 PMC3086437

[B40] Maines TR Szretter KJ Perrone L Belser JA Bright RA Zeng H Tumpey TM Katz JM Pathogenesis of emerging avian influenza viruses in mammals and the host innate immune response Immunol Rev 2008 225 68 84 10.1111/j.1600-065X.2008.00690.x 18837776

[B41] Us D Cytokine storm in avian influenza Mikrobiyol Bul 2008 42 2 365 80 18697437

[B42] Woo PC Tung ET Chan KH Lau CC Lau SK Yuen KY Cytokine profiles induced by the novel swine-origin influenza A/H1N1 virus: implications for treatment strategies J Infect Dis 2010 201 3 346 53 10.1086/649785 20030555 PMC7202468

[B43] Lee SM Gardy JL Cheung CY Cheung TK Hui KP Ip NY Guan Y Hancock RE Peiris JS Systems-level comparison of host-responses elicited by avian H5N1 and seasonal H1N1 influenza viruses in primary human macrophages PLoS One 2009 4 12 e8072 10.1371/journal.pone.0008072 20011590 PMC2788213

[B44] Zhang C Xu Y Jia L Yang Y Wang Y Sun Y Huang L Qiao F Tomlinson S Liu X Zhou Y Song H A new therapeutic strategy for lung tissue injury induced by influenza with CR2 targeting complement inhibitor Virol J 2010 7 30 10.1186/1743-422X-7-30 20144216 PMC2829536

[B45] Shishkina LN Nebol'sin VE Skarnovich MO Kabanov AS Sergeev AA Erdyneeva UB Serova OA Demina OK Agafonov AP Stavskiĭ EA Drozdov IG In vivo efficacy of Ingavirin against pandemic A (H1N1/09)v influenza virus Antibiot Khimioter 2010 55 5-6 32 5 21033472

[B46] Leneva IA Fediakina IT Eropkin MIu Gudova NV Romanovskaia AA Danilenko DM Vinogradova SM Lepeshkin AIu Shestopalov AM Study of the antiviral activity of Russian anti-influenza agents in cell culture and animal models Vopr Virusol 2010 55 3 19 27 20608077

[B47] Fenton RJ Morley PJ Owens IJ Gower D Parry S Crossman L Wong T Chemoprophylaxis of influenza A virus infections, with single doses of zanamivir, demonstrates that zanamivir is cleared slowly from the respiratory tract Antimicrob Agents Chemother 1999 43 11 2642 7 10543741 10.1128/aac.43.11.2642 PMC89537

[B48] Loginova SIa Borisevich SV Maksimov VA Bondarev VP Nebol'sin VE Investigation of prophylactic activity of Ingavirin, a new Russian drug, against grippe A virus (H3N2) Antibiot Khimioter 2008 53 11-12 19 21 19441652

[B49] Loginova SIa Borisevich SV Maksimov VA Bondarev VP Nebol'sin VE Therapeutic efficacy of Ingavirin, a new domestic formulation against influenza A virus (H3N2) Antibiot Khimioter 2008 53 7-8 27 30 19227120

[B50] Leelarasamee A Jongwutiwes U Tantipong H Puthavathana P Siritantikorn S Fulminating influenza pneumonia in the elderly: a case demonstration J Med Assoc Thai 2008 9 924 30 18697395

[B51] Miyamoto D Hasegawa S Sriwilaijaroen N Yingsakmongkon S Hiramatsu H Takahashi T Hidari K Guo CT Sakano Y Suzuki T Suzuki Y Clarithromycin inhibits progeny virus production from human influenza virus-infected host cells Biol Pharm Bull 2008 31 217 22 10.1248/bpb.31.217 18239276

[B52] Zhirnov O Klenk HD Human influenza A viruses are proteolytically activated and do not induce apoptosis in CACO-2 cells Virology 2003 313 198 212 10.1016/S0042-6822(03)00264-2 12951033

[B53] Shneĭder MA Shtil'bans EB Rachkovskaia LA Poltorak VA Antiviral action of carbonyl-conjugated pentaene macrolides Antibiotiki 1983 28 352 7 6309075

[B54] Bermejo-Martin JF Kelvin DJ Eiros JM Castrodeza J Ortiz de Lejarazu R Macrolides for the treatment of severe respiratory illness caused by novel H1N1 swine influenza viral strains J Infect Dev Ctries 2009 3 3 159 61 19759469 10.3855/jidc.18

[B55] Weinbroum AA Kluger Y Ben Abraham R Shapira I Karchevski E Rudick V Lung preconditioning with N-acetyl-L-cysteine prevents reperfusion injury after liver no flow-reflow: a dose-response study Transplantation 2001 71 2 300 6 10.1097/00007890-200101270-00023 11213077

[B56] Moldéus P Cotgreave IA Berggren M Lung protection by a thiol-containing antioxidant: N-acetylcysteine Respiration 1986 suppl 1 31 42 10.1159/000195086 3809741

[B57] Adamson WE Maddi S Robertson C McDonagh S Molyneaux PJ Templeton KE Carman WF 2009 pandemic influenza A(H1N1) virus in Scotland: geographically variable immunity in Spring 2010, following the winter outbreak Euro Surveill 2010 15 24 pii: 19590 20576237

